# Supplementary material for: Improved emission of SiV diamond color centers embedded into concave plasmonic core-shell nanoresonators
Source: Sci Rep. 2017 Oct 23;7:13845. doi: 10.1038/s41598-017-14227-w (PMC5653742; doi:10.1038/s41598-017-14227-w)
Supplement: Supplementary file 1 — Supplementary Material [file 41598_2017_14227_MOESM1_ESM.pdf]

Improved emission of SiV diamond color centers embedded into concave plasmonic core-shell nanoresonators

András Szenes[1], Balázs Bánhelyi[2], Lóránt Zs. Szabó[1], Gábor Szabó[1], Tibor Csendes[2], Mária Csete[1,*]

[1]Department of Optics and Quantum Electronics, University of Szeged, Dóm tér 9, Szeged, H-6720, Hungary.
[2]Institute of Informatics, University of Szeged, Árpád tér 2, Szeged, H-6720, Hungary

*mcsete@physx.u-szeged.hu, *+36-62-544654**

Sensitivity study and experimental feasibility

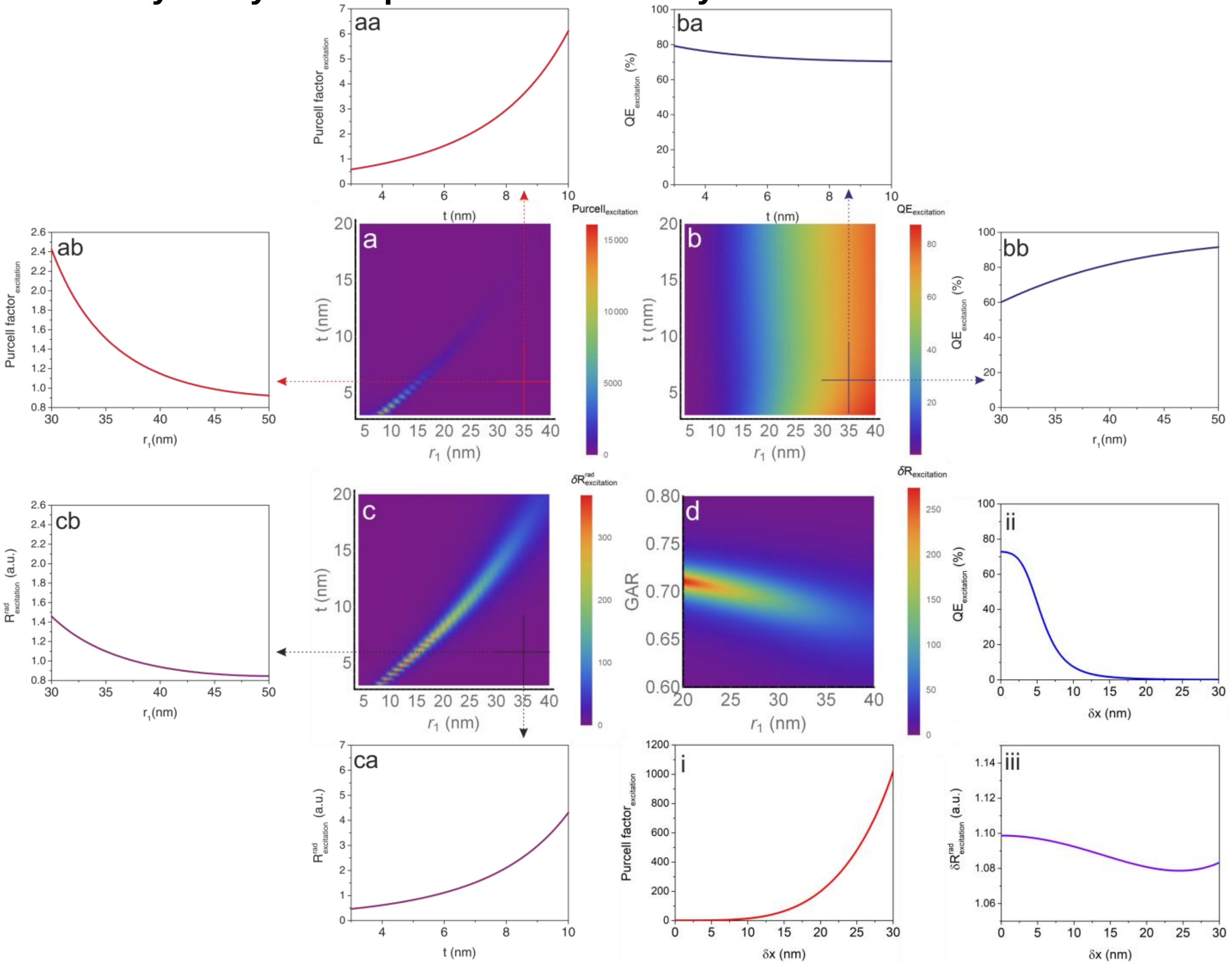

Figure S1: Optical response of CSCS configuration above the core radius (r_1) – shell thickness (t) parameter space. (a) *Purcell factor* (b) *QE* (c) $\delta R^{rad}_{excitation}$ radiative rate enhancement vs. core radius and shell thickness (d) $\delta R^{rad}_{excitation}$ radiative rate enhancement vs. core radius and generalized aspect ration (*GAR)* at SiV excitation wavelength. Insets: projections of the 2D parameter space (aa) *Purcell factor* vs. shell thickness at optimal core radius [35 nm] and (ab) *Purcell factor* vs. core radius at optimal shell thickness [5.96 nm], (ba) *QE* vs. shell thickness at optimal core radius [35 nm] and (bb) *QE* vs. core radius at optimal shell thickness [5.96 nm], (ca) $\delta R^{rad}_{excitation}$ radiative rate enhancement vs. shell thickness at optimal core radius [35 nm] and (cb) $\delta R^{rad}_{excitation}$ radiative rate enhancement vs. core radius at optimal shell thickness [5.96 nm] at excitation wavelength. (i-iii) *Purcell factor*, *QE*, $\delta R^{rad}_{excitation}$ radiative rate enhancement vs. dipole displacement at SiV excitation wavelength.

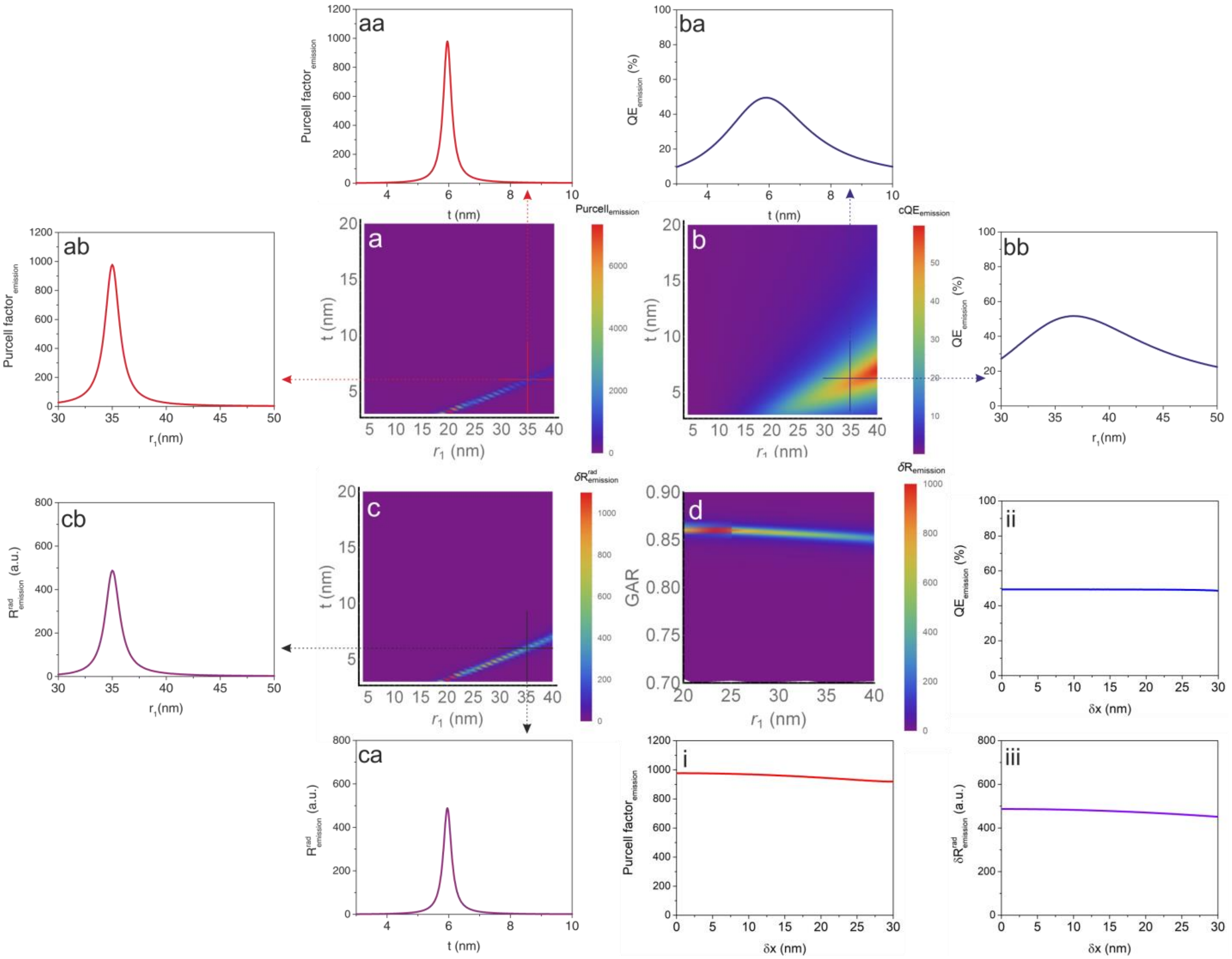

Figure S2: Optical response of CSCS configuration above the core radius (r_1) – shell thickness (t) parameter space. (a) *Purcell factor* (b) *QE* (c) $\delta R^{rad}_{emission}$ radiative rate enhancement vs. core radius and shell thickness, (d) $\delta R^{rad}_{emission}$ radiative rate enhancement vs. core radius and generalized aspect ration (*GAR)* at SiV emission wavelength. Insets: projections of the 2D parameter space (aa) *Purcell factor* vs. shell thickness at optimal core radius [35 nm] and (ab) *Purcell factor* vs. core radius at optimal shell thickness [5.96 nm], (ba) *QE* vs. shell thickness at optimal core radius [35 nm] and (bb) *QE* vs. core radius at optimal shell thickness [5.96 nm], (ca) $\delta R^{rad}_{emission}$ radiative rate enhancement vs. shell thickness at optimal core radius [35 nm] and (cb) $\delta R^{rad}_{emission}$ radiative rate enhancement vs. core radius at optimal shell thickness [5.96 nm] at emission wavelength. (i-iii) *Purcell factor*, *QE*, $\delta R^{rad}_{emission}$ radiative rate enhancement vs. dipole displacement at SiV emission wavelength.

Only one single resonance and a corresponding Purcell peak appears in the inspected geometrical parameter interval both at the SiV excitation and at the SiV emission wavelengths (Fig. S1a and S2a). The resonance energy is dependent mainly (exclusively) on the generalized aspect ratio at the excitation (emission) (Fig. S1d and S2d), only slight deflection is observable on the curve corresponding to the $\delta R^{rad}_{excitation/emission}$ radiative rate enhancement above the *GAR* generalized aspect ratio and r_1 core radius parameter space. The *cQE* corrected quantum efficiency can be high only at radiative resonances (Fig. S2b).

In this study all results are presented with 0.1% accuracy according to the high numerical accuracy of the FEM computations. Via current experimental methodologies such high precision can be just approximated, therefore the effect of geometrical uncertainty on the fluorescence enhancement was inspected. The sensitivity of the P_x factor to the geometrical parameters, e.g. core radius, shell thickness, dipole displacement, has been analyzed for the optimized CSCS system (Fig. S3).

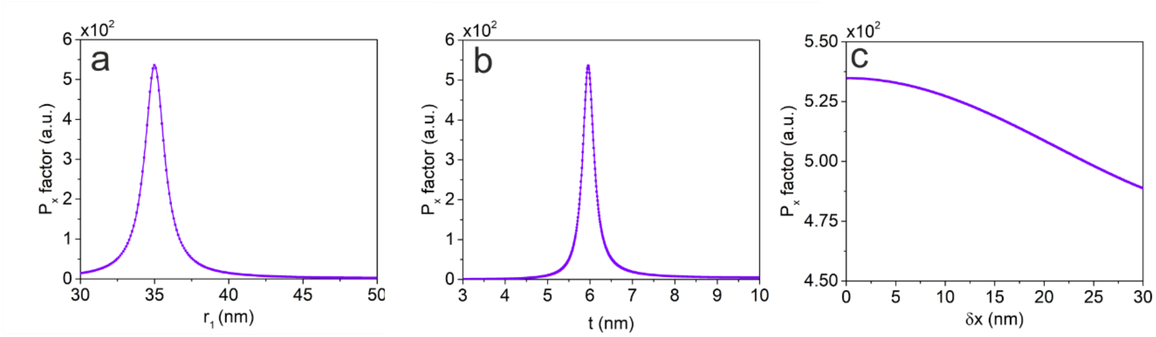

Figure S3: Dependence of the complete CSCS fluorescence enhancement (P_x factor) on geometric parameters: (a) diamond core radius, (b) silver shell thickness and (c) dipole displacement.

The sensitivity study revealed that the P_x factor remains in the same order of magnitude, if the core radius is within the +/-2.5 nm interval of the optimal value, while the shell thickness has to be in the +/-0.5 nm interval to meet this criterion. These allowed intervals correspond to 7.1% and 8.4% uncertainty in the core radius and metal shell, respectively. In contrast, the sensitivity to the dipole displacement is significantly smaller, namely only 8.6% P_x factor decrease is caused by 30 nm displacement, which corresponds to 85.7% discrepancy from the optimal position.

Current nanofabrication methods make possible to prepare nanodiamonds down to the order of 2-5 nm [S1]. Representative experimental results have been already published on successful covering of 200 nm nanodiamonds by a gold shell having a thickness of 20 nm via a modified seed-growth method [S2]. Moreover, it is possible to cover dielectric nanoparticles by silver, e.g. by anchoring 2-4 nm Ag nanocrystals, 2-10 nm reproducible but non-continuous silver layer was coated onto silica surfaces [S3]. Although, discontinuity of the chemically prepared shells could cause discrepancies with respect to the optimized systems, inspection of such non-optimal structures was out of scope of the present study.

Based on the previously described experimental procedures and the possibility of nanodiamond surface modifications, silver-coated nanodiamonds can be prepared as well [S2]. It is known that the surface charge of the nanodiamonds strongly influences the type of the reduction process, e.g. negatively charged surface promotes the binding of positively charged silver ions onto the surface [S4, S5]. The reduction of Ag+ ions with hydroxylamine hydrochloride can be carried out in presence of nanodiamonds in aqueous dispersion. Other possible process is the reduction of Ag+ ions using the well-known photoreduction [S6].

The special rod-like particles can be fabricated by evaporating than cutting the diamond nanowires fabricated via e-beam lithography [S7]. Fabrication of ellipsoidal nanodiamond can be realized by etching and ellipsoidal nanoparticles can be also covered by continuous films seeded via metal nanocrystals [S8].

Comparative study on coupled SiV color center - concave nanoresonator systems optimized with different criteria

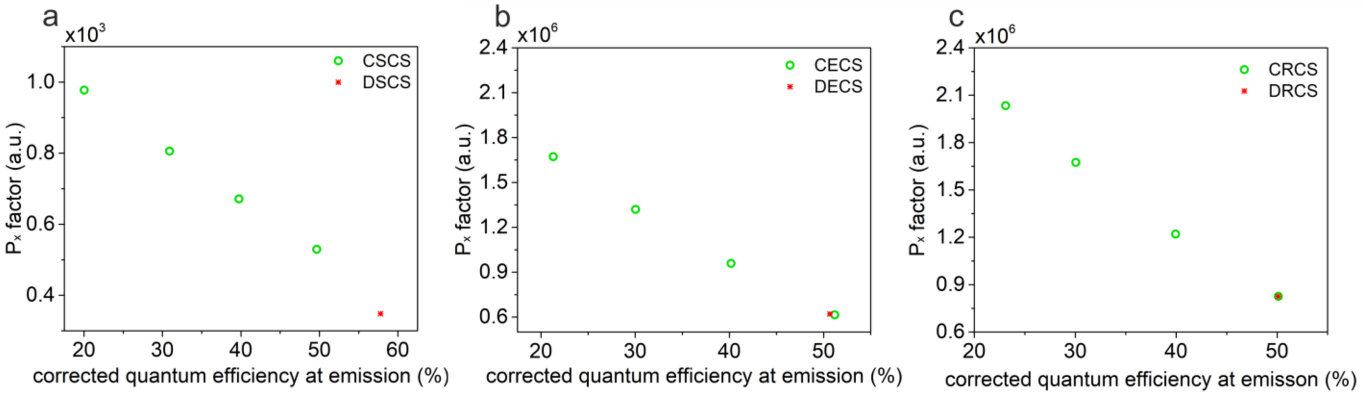

Figure S4: Total fluorescence enhancement (P_x *factor*) of (a) spherical, (b) ellipsoidal and (c) rod-shaped concave core-shell nanoresonators containing a centralized dipole and optimized with 50, 40, 30 and 20% *cQE* criteria. The P_x *factor* of the optimized configurations consisting of a decentralized dipole corresponding to *cQE*=50% are also shown by red stars.

The optimization results show that in all optimized configurations at the emission wavelength the $\delta R^{rad}_{emission}$ radiative rate enhancement of SiV color center decreases by increasing the criterion regarding the corrected quantum efficiency (Fig. S4).

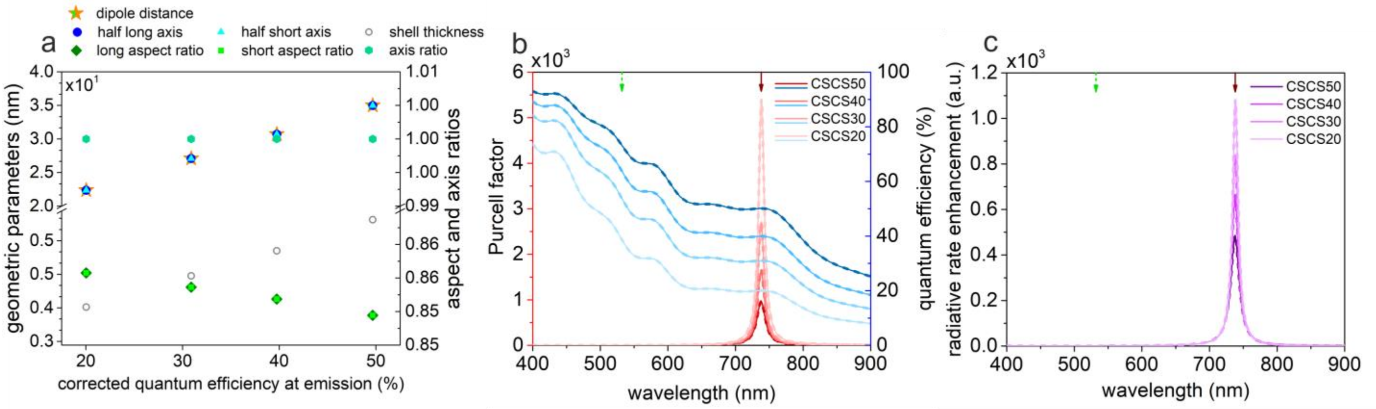

Figure S5: Parameters and optical response of CSCS optimized with 50, 40, 30 and 20% *cQE* criteria. (a) Tendency of optimal geometric parameters, axis and generalized aspect ratios vs. required *cQE*, (b) *Purcell factor* and *QE* spectra and (c) δR^{rad} radiative rate enhancement spectra of spherical core-shell nanoresonators containing a centralized dipole in excitation (dashed lines) and emission (solid lines) configuration.

In case of CSCS the achievable *cQE* is governed by the distance between the emitter and the absorbing metal, as well as by the metal shell thickness. Namely, the higher the desired *cQE* is, the larger is the diamond core radius, and surprisingly the *t* shell thickness increases as well. As a result, the total spherical core-shell nanoresonator size increases nearly linearly in the quasistatic limit by increasing the *cQE* criterion (Fig. S5a).

The position of the sole visible resonance peak is mainly determined by the *GAR* aspect ratio of the concave core-shell nanoresonator (Table S1). In all optimized coupled systems the symmetric bonding dipolar mode is tuned to the emission wavelength. Accordingly, the *GAR* of nanoshells are nearly the same, only a slight *GAR* decrease is observable as the *cQE* criterion increases (Fig. S5a). However, the larger core-shell nanoresonator size leads to a weaker resonance as the *Purcell factor* spectra show (Fig. S5b). As a consequence, the $\delta R^{rad}_{emission}$ radiative rate enhancement at the emission decreases by increasing the *cQE* criterion (Fig. S5c).

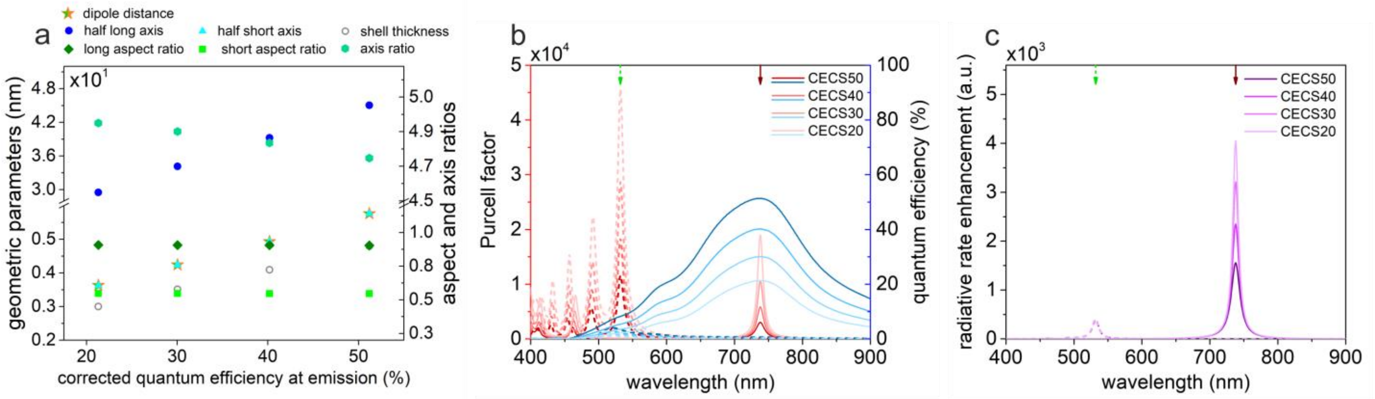

Figure S6: Parameters and optical response of CECS optimized with 50, 40 30 and 20% *cQE* criteria. (a) Tendency of optimal geometric parameters, axis and generalized aspect ratios vs. required *cQE*, (b) *Purcell factor* and *QE* spectra and (c) δR^{rad} radiative rate enhancement spectra of ellipsoidal core-shell nanoresonators containing a centralized dipole in excitation (dashed lines) and emission (solid lines) configuration.

The interdependence of the geometrical parameters and the resonance energy is more complex in case of concave ellipsoidal core-shell nanoresonators. Generally, it can be concluded again that larger *cQE* requires larger emitter-metal distance (Fig. S6a). To increase the dipole distance one should increase the short axis of the ellipsoid as well. To maintain the transversal and longitudinal resonances at SiV excitation and emission wavelengths, the ratio of long and short axes should be conserved. Accordingly, the higher the desired *cQE* is, the larger is the diamond core short and long axis, and surprisingly the *t* shell thickness increases as well. As a result, the total ellipsoidal core-shell nanoresonator size increases similarly to CSCS.

In the *GARs* corresponding to the short and long axis of nanoshells only a slight decrease is observable, as the *cQE* criterion increases (Table S1). Moreover, not only the aspect ratio but the axes lengths themselves influence the energies of the resonance peaks. The larger *cQE* at emission requires larger metal-emitter distance, accordingly larger short and long axis. A slightly smaller long-to-short axis ratio is preferred, as the *cQE* increases.

Similarly to CSCS nanoresonators, the higher is the *cQE*, the lower is the *Purcell factor* and the $\delta R^{rad}_{excitation/emission}$ radiative decay rate enhancement both at the excitation and emission (Fig. S6b and c). In CECS by decreasing the *cQE* criterion from 50% through 20%, the relative radiative rate enhancement increase is insignificant at the excitation, while at the emission the increase is more than 2-fold.

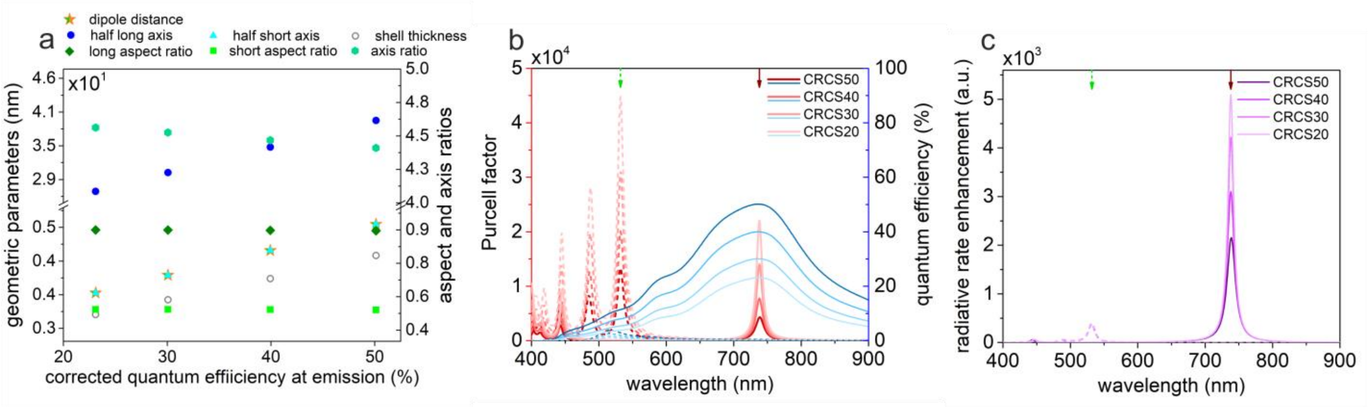

Figure S7: Parameters and optical response of CRCS optimized with 50, 40, 30 and 20% *cQE* criteria. (a) Tendency of optimal geometric parameters, axis and generalized aspect ratios vs. required *cQE*, (b) *Purcell* factor and *QE* spectra and (c) δR^{rad} radiative rate enhancement spectra of rod-like core-shell nanoresonator containing a centralized dipole in excitation (dashed lines) and emission (solid lines) configuration.

In case of CRCS the tendencies are the same as in CECS, namely to achieve higher *cQE* larger dipole distance is required, which implies appropriatley larger short and long axis, and maintainance of the optimal decreasing axis lenght ratio (Fig. S7a). Surprisingly the thickness of the metal shell increases as well. In the *GARs* corresponding to the short and long axis of nanoshells only a slight decrease is observable. The rod-shaped concave core-shell nanoresonators show a *Purcell factor* and $\delta R^{rad}_{excitation/emission}$ radiative rate enhancement characteristics and *QE* spectra similar to those of CECS (Fig. S7b and c). Although, their excitation rate enhancements are almost identical, a major difference between CRCS and CECS is that the CRCSs have significantly larger *Purcell factor* at the emission. Despite the smaller dipole distance from the metal, at the same *cQE* larger $\delta R^{rad}_{emission}$ radiative rate enhancement is achieved in case of CRCSs, due to the efficient far-field coupling via rod-shaped nano-antennas. The highest $2.03 \cdot 10^6$ fluorescence enhancement among the inspected concave plasmonic nanoresonators was achieved via rod-shaped cores embedded into silver nanoshell, when the criterion regarding the minimum *cQE* was set to 20%.

References

[S1] Vlasov, I. I., et al. Molecular-sized fluorescent nanodiamonds. Nat. Nanotechnol. 9, 54-58 (2014).

[S2] Minati, L., et al. Synthesis of novel nanodiamonds–gold core shell nanoparticles. Diam. Relat. Mater. 53, 23-28 (2015).

[S3] Huang, L., et al. Plasmonic silver nanoshells for drug and metabolite detection. Nat. Commun. 8, 220 (2017).

[S4] Paci, J. T., et al. Understanding the Surfaces of Nanodiamonds. J. Phys Chem. C, 117, 17256-17267 (2013).

[S5] Gibson, N., et al. Colloidal stability of modified nanodiamond particles. Diam. Relat. Mater. 18, 620-626 (2009).

[S6] Sato-Berrú, R., et al. Silver nanoparticles synthesized by direct photoreduction of metal salts. Application in surface-enhanced Raman spectroscopy. J. Raman Spectrosc. 40, 376-380 (2009).

[S7] Hausmann, B. J. M., et al. Single-color centers implanted in diamond nanostructures. New J. Phys. 13, 045004 (2011).

[S8] Wang, H., et al. Nanorice: A Hybrid Plasmonic Nanostructure. Nano Lett. 6, 827-832 (2006).

	excitation				emission					geometry								
	**criterion***	***Purcell factor***	***QE* (%)**	**δR^{rad} (a.u.)***	**criterion***	***Purcell factor***	***cQE* (%)***	**δR^{rad} (a.u.)**	**P_x *factor* (a.u.)**	***d* (nm)**	**r_1 (nm)**	**r_2 (nm)**	***t* (nm)**	**GAR_1**	**GAR_2**	***AXR***	**δx (nm)**	**δy (nm)**
**DSCS50**	1	3.13E+02	3.22E-01	1.01E+00	50	5.55E+02	5.75E+01	3.19E+02	3.21E+02	9.59E+00	3.92E+01	3.92E+01	6.80E+00	8.52E-01	8.52E-01	1.00E+00	2.96E+01	0.00E+00
**CSCS50**	1	1.50E+00	7.32E+01	1.10E+00	50	9.71E+02	4.97E+01	4.82E+02	5.29E+02	3.50E+01	3.50E+01	3.50E+01	5.96E+00	8.54E-01	8.54E-01	1.00E+00	0.00E+00	0.00E+00
**CSCS40**	1	1.60E+00	6.37E+01	1.02E+00	40	1.66E+03	3.98E+01	6.60E+02	6.71E+02	3.07E+01	3.07E+01	3.07E+01	5.13E+00	8.57E-01	8.57E-01	1.00E+00	0.00E+00	0.00E+00
**CSCS30**	0	1.79E+00	5.38E+01	9.64E-01	30	2.71E+03	3.09E+01	8.36E+02	8.06E+02	2.71E+01	2.71E+01	2.71E+01	4.46E+00	8.59E-01	8.59E-01	1.00E+00	0.00E+00	0.00E+00
**CSCS20**	0	2.34E+00	3.87E+01	9.03E-01	20	5.40E+03	2.00E+01	1.08E+03	9.78E+02	2.24E+01	2.24E+01	2.24E+01	3.62E+00	8.61E-01	8.61E-01	1.00E+00	0.00E+00	0.00E+00

	excitation				emission					geometry								
	**criterion***	***Purcell factor***	***QE* (%)**	**δR^{rad} (a.u.)***	**criterion***	***Purcell factor***	***cQE* (%)***	**δR^{rad} (a.u.)**	**P_x *factor* (a.u.)**	***d* (nm)**	**r_1 (nm)**	**r_2 (nm)**	***t* (nm)**	**GAR_1**	**GAR_2**	***AXR***	**δx (nm)**	**δy (nm)**
**DECS50**	1	1.19E+04	3.30E+00	3.92E+02	50	3.13E+03	5.06E+01	1.58E+03	6.20E+05	3.32E+00	4.48E+01	5.72E+00	4.76E+00	9.04E-01	5.46E-01	4.73E+00	-4.29E+00	2.37E+00
**CECS50**	1	1.16E+04	3.42E+00	3.96E+02	50	3.03E+03	5.12E+01	1.55E+03	6.15E+05	5.76E+00	4.51E+01	5.76E+00	4.79E+00	9.04E-01	5.46E-01	4.72E+00	0.00E+00	0.00E+00
**CECS40**	1	1.84E+04	2.21E+00	4.06E+02	40	5.86E+03	4.01E+01	2.35E+03	9.54E+05	4.93E+00	3.92E+01	4.93E+00	4.09E+00	9.06E-01	5.46E-01	4.80E+00	0.00E+00	0.00E+00
**CECS30**	1	2.87E+04	1.43E+00	4.11E+02	30	1.07E+04	3.00E+01	3.21E+03	1.32E+06	4.24E+00	3.42E+01	4.24E+00	3.51E+00	9.07E-01	5.47E-01	4.86E+00	0.00E+00	0.00E+00
**CECS20**	1	4.57E+04	9.04E-01	4.13E+02	20	1.90E+04	2.13E+01	4.05E+03	1.67E+06	3.63E+00	2.95E+01	3.63E+00	3.00E+00	9.08E-01	5.47E-01	4.90E+00	0.00E+00	0.00E+00

	excitation				emission					geometry								
	**criterion***	***Purcell factor***	***QE* (%)**	**δR^{rad} (a.u.)***	**criterion***	***Purcell factor***	***cQE* (%)***	**δR^{rad} (a.u.)**	**P_x *factor* (a.u.)**	***d* (nm)**	**r_1 (nm)**	**r_2 (nm)**	***t* (nm)**	**GAR_1**	**GAR_2**	***AXR***	**δx (nm)**	**δy (nm)**
**DRCS50**	1	1.32E+04	2.94E+00	3.88E+02	50	4.27E+03	5.03E+01	2.15E+03	8.34E+05	5.37E+00	3.92E+01	5.38E+00	4.55E+00	8.96E-01	5.42E-01	4.40E+00	0.00E+00	1.00E-02
**CRCS50**	1	1.34E+04	2.90E+00	3.88E+02	50	4.27E+03	5.00E+01	2.14E+03	8.29E+05	5.36E+00	3.92E+01	5.36E+00	4.55E+00	8.96E-01	5.41E-01	4.41E+00	0.00E+00	0.00E+00
**CRCS40**	1	2.01E+04	1.96E+00	3.93E+02	40	7.77E+03	3.99E+01	3.10E+03	1.22E+06	4.67E+00	3.46E+01	4.67E+00	3.94E+00	8.98E-01	5.43E-01	4.47E+00	0.00E+00	0.00E+00
**CRCS30**	1	3.11E+04	1.28E+00	3.97E+02	30	1.40E+04	3.01E+01	4.22E+03	1.67E+06	4.03E+00	3.02E+01	4.03E+00	3.39E+00	8.99E-01	5.43E-01	4.53E+00	0.00E+00	0.00E+00
**CRCS20**	1	4.48E+04	8.91E-01	3.99E+02	20	2.21E+04	2.31E+01	5.10E+03	2.03E+06	3.57E+00	2.70E+01	3.57E+00	3.00E+00	9.00E-01	5.43E-01	4.56E+00	0.00E+00	0.00E+00

Table S1: Optical response and geometric parameters of optimized SCS, ECS and RCS configurations.
QE: quantum efficiency, $\delta R^{rad}_{excitation/emission}$: radiative rate enhancement, *cQE*: corrected quantum efficiency, P_x *factor*: total fluorescence rate enhancement ($\delta R^{rad}_{excitation} \cdot \delta R^{rad}_{emission}$), *d*: minimum dipole distance from metal nanoshell, r_1: radius of diamond core along the long axis, r_2: radius of diamond core along the short axis, *t*: silver shell thickness, GAR_1: generalized aspect ratio along the long axis ($r_1/(r_1+t)$), GAR_2: generalized aspect ratio along the long axis ($r_2/(r_2+t)$), *AXR*: axis ratio ($(r_1+t)/(r_2+t)$), δx: dipole decentralization along *x* axis, δy: dipole decentralization along *y* axis.
